# The Antiviral Activity of Interferon-Induced Transmembrane Proteins and Virus Evasion Strategies

**DOI:** 10.3390/v16050734

**Published:** 2024-05-06

**Authors:** Jingjing Wang, Yuhang Luo, Harshita Katiyar, Chen Liang, Qian Liu

**Affiliations:** 1 Institute of Parasitology, McGill University, Ste Anne de Bellevue, QC H9X 3V9, Canada; jingjing.wang@mail.mcgill.ca (J.W.); yuhang.luo@mail.mcgill.ca (Y.L.); 2 McGill Center for Viral Diseases, Lady Davis Institute, Montreal, QC H3T 1E2, Canada; harshita.katiyar@mail.mcgill.ca (H.K.); chen.liang@mcgill.ca (C.L.); 3 Division of Experimental Medicine, McGill University, Montreal, QC H4A 3J1, Canada

**Keywords:** IFITMs, antiviral activity, virus and cell membrane fusion, viral glycoproteins, virus evasion, immune modulation

## Abstract

Interferons (IFNs) are antiviral cytokines that defend against viral infections by inducing the expression of interferon-stimulated genes (ISGs). Interferon-inducible transmembrane proteins (IFITMs) 1, 2, and 3 are crucial ISG products and members of the CD225 protein family. Compelling evidence shows that IFITMs restrict the infection of many unrelated viruses by inhibiting the virus–cell membrane fusion at the virus entry step via the modulation of lipid composition and membrane properties. Meanwhile, viruses can evade IFITMs’ restrictions by either directly interacting with IFITMs via viral glycoproteins or by altering the native entry pathway. At the same time, cumulative evidence suggests context-dependent and multifaceted roles of IFITMs in modulating virus infections and cell signaling. Here, we review the diverse antiviral mechanisms of IFITMs, the viral antagonizing strategies, and the regulation of IFITM activity in host cells. The mechanisms behind the antiviral activity of IFITMs could aid the development of broad-spectrum antivirals and enhance preparedness for future pandemics.

## 1. Introduction

Interferons (IFNs) are produced following pathogen recognition by membrane-bound or cytosolic receptors, such as Toll-like receptors and RIG-I-like receptors. There are three families of interferons, type I (e.g., IFNα), type II (e.g., IFNγ), and type III (e.g., IFNλ). IFNs bind to IFN receptors and activate the transcription of interferon-stimulated genes (ISGs), which act against pathogens and regulate innate and adaptive immunity. Interferon-induced transmembrane (IFITM) genes are typical ISGs, although they can be constitutively expressed in some cells, like stem cells and barrier epithelial cells [1]. The discovery of the antiviral activity of IFITMs was dated back to the early 1990s, when IFITM1 was found to inhibit the replication of vesicular stomatitis virus (VSV) [2]. Expanding upon this observation, one subsequent study identified IFITMs as host factors restricting H1N1 influenza virus infection by siRNA screening in 2009 [3]. These discoveries stimulated the research on the antiviral activity and the underlying mechanisms of IFITMs. A large body of in vitro and in vivo work has been conducted to dissect the role of IFITMs at the early steps of viral infection through employing live viruses, pseudotyped viruses, IFITM overexpression and knockdown cells, and animal models. Here, we review the various mechanisms by which IFITMs modulate viral infection and the viral strategies to escape and/or hijack IFITMs.

## 2. The Broad-Spectrum Antiviral Activity of IFITMs

The human *ifitm* locus is approximately 18 kb long and located on chromosome 11. It comprises five genes, *ifitm1*, *ifitm2*, *ifitm3*, *ifitm5*, and *ifitm10*, among which IFITM1, 2, and 3 are ubiquitously expressed in human tissues and stimulated by type I and type II IFNs, while the IFITM5 and 10 are not induced by IFNs [4]. As ISGs, IFITM1, 2, and 3 have an interferon-stimulated response element (ISRE) in their promoters in addition to a gamma-activated sequence (GAS) [4]. Most vertebrates have two or more *ifitm* genes and frequent gene duplications have been observed on immunity-related *ifitm* (IR-*ifitm*) genes in primates and rodents [5]. A comparative genomic analysis reveals that *ifitm*3 is the oldest *ifitm* gene with known antiviral activities [6]. Moreover, *ifitm*2 emerged relatively recently in human, chimpanzee, and gorilla [6]. The ongoing duplication of *ifitm* genes in different species suggests a positive selection pressure and is expected to provide advantage to species survival [6]. The *ifitm* gene duplication also leads to the generation of pseudogenes. Xiao et al. recently reported that the long noncoding *ifitm3* pseudogene (*ifitm*4p) acts as a competing endogenous RNA that binds to the microRNA miR-24-3p, which represses mRNAs of IFITM1, 2, and 3, thereby regulating the mRNA levels of IFITM1, 2, and 3 and their virus restriction ability [7]. Overall, gene duplications expand the repertoires of the *ifitm* gene family and indicate a positive selection in IFITM evolution that is consistent with their role in antiviral immunity.

**Figure 1:**
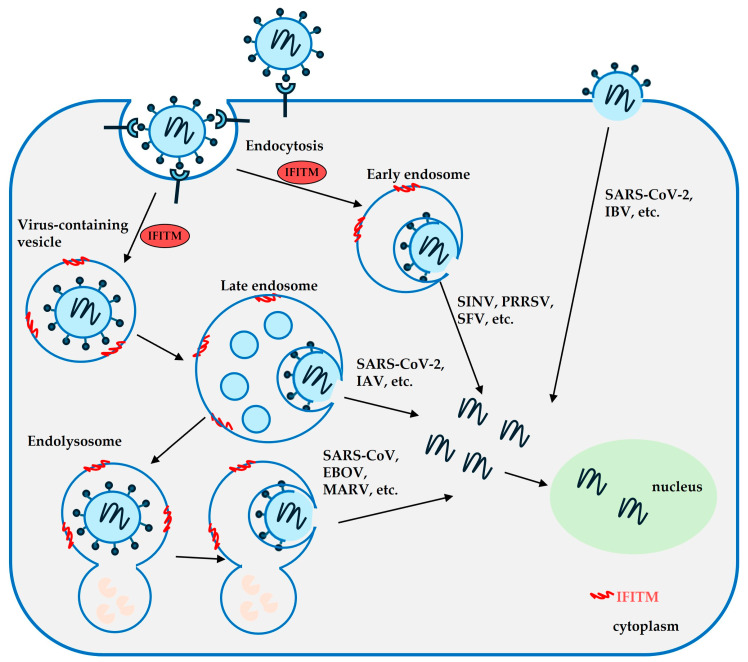
Antiviral mechanism of interferon-inducible transmembrane proteins IFITMs. IFITMs inhibit virus entry at different steps.

IFITMs inhibit a wide range of RNA and DNA viruses (Table 1) by mechanisms of preventing the fusion of viral and cellular membranes during the initial stage of virus infection (Figure 1). IFITMs also inhibit the infection of non-enveloped viruses by interfering with endosome function. For example, IFITM3 alters the function of late endosomal compartments, thus slowing rotavirus to escape from endosomes and limiting infection [8]. A large number of viruses restricted by IFITMs are clinically important, pathogenic viruses, such as influenza A virus (IAV) [3], Ebola and Marburg viruses [9], respiratory syncytial virus (RSV) [10], Human Immunodeficiency Virus-1 (HIV-1) [11,12], and Zika virus (ZIKV) [13], illuminating the importance of IFITMs in protecting humans from viral diseases. IFITMs can inhibit viral infection through multiple pathways and mechanisms, such as modulating virus-cell membrane fusion [14,15,16], targeting viral glycoproteins and host receptors [11,17,18], and altering the key factors in the entry pathway (e.g., endosomes) [19,20].

The importance of IFITMs in antiviral defense in vivo has been well addressed in studies using IFITM3 knockout (KO) mice and mice lacking the entire IFITM locus (IFITMdel). Using these mouse models, IFITM3 was shown to prevent severe pathology upon virus infections in mice, including SARS-CoV-2 [54], IAV (H1N1 and H3N2) [55,56], WNV [57], CHIKV, Venezuelan equine encephalitis virus [58], RSV [59], and CMV [60]. For example, the IFITM3 KO mice are more susceptible to H1N1 and H3N2 IAV infection than WT mice, manifesting more severe weight loss, greater mortality, higher virus titers in the lungs, systemic lymphopenia, and drastic production of pro-inflammatory cytokines in the lungs [56]. Interestingly, although IFITMdel mice were more susceptible to IAV infection than mice carrying intact IFITM gene locus, IFITMdel mice did not manifest more severe infections than IFITM3 KO mice, implying that the IFITM3 protein is critical in restricting IAV in mice [56]. SARS-CoV-2 infection resulted in greater weight loss and lethality in IFITM3 KO mice along with higher viral titers in the lungs, elevated levels of inflammatory cytokines, immune cell infiltration, and histopathology as compared to WT mice [54]. A mechanistic study revealed that IFITM3 expression is selectively maintained in the anti-influenza CD8+ resident memory T cells in the lungs so that these cells are resistant to IAV infection and capable of protecting against subsequent infections [61]. In addition to the lungs, IFITM3 has also been shown to protect the heart against severe IAV infection since the IFITM3 KO mice develop aberrant cardiac electrical activity following infection with the highly pathogenic A/PR/8/34 H1N1 virus [62]. Similarly, IFITM3 was capable of restricting RSV in vivo either by directly restricting RSV infection or by controlling pathogenesis [59].

In support of viral infection results in IFITM KO mice, multiple studies have shown that single nucleotide polymorphisms (SNPs) in IFITM3 are associated with the severity of IAV and SARS-CoV-2 infections. Everitt et al. reported that the substitution of a majority T allele by a minority C allele of SNP rs12252 was associated with hospitalization during the pandemic H1N1/09 or seasonal influenza infections in 2009–2010 [55]. This substitution codes for the IFITM3 variant lacking the first 21 amino acids at the N-terminus (NΔ21) due to alternative splicing of the IFITM3 mRNA transcript. Further in vitro experiments show that the NΔ21 protein fails to restrict the replication of several virulent influenza viral strains [55]. However, the alternative splicing hypothesis is challenged when the alternative splicing transcript of IFITM3 rs12252-C SNP was not detected in another study [63]. The association between IFITM3 rs12252 and IAV infection severity was not observed in a multicenter cohort of US children admitted to the intensive care unit [64]. Similarly, Kim et al. did not observe a significant difference in the genotype distribution of the rs12252 SNP between the healthy and 2009 H1N1 IAV-infected populations in Korea [65]. Nonetheless, IFITM3 rs12252 was linked to severity and mortality in SARS-CoV-2-infected patients during the COVID-19 pandemic. Patients with the IFITM3 rs12252 (C) allele had a higher risk of COVID-19 mortality than those with the T allele in Chinese and Caucasian populations [66,67,68]. In comparison, a third G allele of IFITM3 rs12252 was significantly associated with hospitalization and mortality in COVID-19 patients in a large Arab population, particularly in the younger population [69]. A second IFITM3 SNP (rs34411844-A) in the IFITM3 promotor was associated with severe IAV infections and death in African–American and European populations. The rs344118844-A SNP downregulates IFITM3 protein and mRNA levels by disrupting the CTCF binding to the IFITM3 promoter [70]. A total of eight IFITM1 SNPs have been reported to date, and the substitution of G to A at rs77537847 may be associated with susceptibility to ulcerative colitis in Korea [71], but no evidence shows association between IFITM1 SNPs and the susceptibility to 2009 H1N1 IAV infection [72].

## 3. The Structure and Post-Translational Modifications of IFITM Proteins

**Figure 2:**
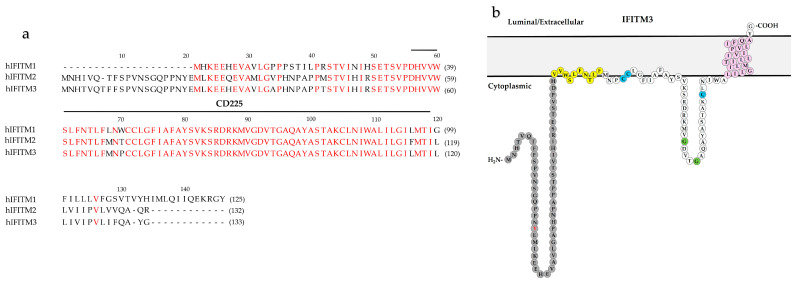
The sequence and membrane topology of IFITMs. (**a**) Alignment of human IFITM1, 2, and 3. CD225 domain is labeled (IFITM1: residues 35–97; IFITM2: residues 55–28; IFITM3: residues 56–129); red letters: conserved amino acid residues. (**b**) The membrane topology of IFITM3. Grey: variable N-terminal domain (residues 1–57); yellow: the amphipathic helix domain; blue: the S-palmitoylated cysteine residues; green: the glycine residues in GxxxG domain; red letter: 20 Tyrosine (20Y); pink: transmembrane domain (Adapted from Rahman et al. [75]).

IFITMs belong to the dispanin/CD225 family of proteins and are characterized by a similar structure (Figure 2a). Human IFITM3 consists of a variable N-terminal domain (NTD; residues 1–57), an intramembrane domain (IMD; also known as IM1, residues 58–80), a cytoplasmic intracellular loop (CIL; residues 81–107), and a transmembrane domain (TMD; also known as TM2, residues 108–133). Although predicted to have multiple membrane topologies, IFITM3 is recognized as a type II transmembrane protein with a C-terminus facing the lumen/extracellular environment, an intramembrane segment in the membrane leaflet, and a transmembrane helix with its N-terminus exposed to the cytoplasm, as detected by NMR [73,74] (Figure 2b).

Post-translational modifications (PTMs) regulate IFITMs trafficking and localization. The palmitoylation/depalmitoylation cycle, a covalent fatty acid modification on cysteine residues, is critical for the trafficking, localization, and function of many membrane-associated proteins. IFITM3 is S-palmitoylated on membrane-proximal cysteine residues (71, 72, and 105) [76,77]. These cysteine residues are conserved among the IFITM protein family in vertebrates [75,76]. Early studies show that alanine mutants of the S-palmitoylated cysteine residues in IFITM3 lead to the loss of antiviral activity against influenza virus [76]. A gain-of-function study showed that the site-specific lipidation of cysteine 72 enhances the antiviral activity of IFITM3 by modulating its conformation and interaction with lipid membranes [78]. S-palmitoylation of IFITM3 can facilitate its binding to cholesterol in cells [79], although IFITM3 partitions to the lipid-disordered phase in lipid vesicles in vitro [80]. S-palmitoylation on Cysteine 72 of IFITM3 regulates the trafficking of IFITM3-positive vesicles toward the IAV-bearing vesicles in HeLa cells [19]. The palmitoyl acyltransferases (PATs) with conserved DHHC amino acid motifs are responsible for the S-palmitoylation of IFITM3, and a single PAT is sufficient to increase the antiviral activity of IFITM proteins [81]. A depalmitoylase, α/β-hydrolase domain-containing 16A (ABHD16A) is identified as a critical enzyme that catalyzes the depalmitoyl reaction of the S-palmitoylated IFITMs and thus decreases the antiviral activity of IFITMs [82]. The N-terminal region of human IFITM3 contains a 20YEML23 sorting signal that localizes IFITM3 to the endosome [83,84,85]. The phosphorylation or mutation of Y20 redistributes IFITM3 from endosomes to the plasma membrane and results in decreased antiviral activity against IAV but not HIV-1 [83,84]. The endosomal localization is also regulated by ubiquitination. IFITM3 is polyubiquitinated at lysine-48 and lysine-63 [76]. Poly-ubiquitination of IFITM3 dysregulates the endosomal localization of IFITM3 and its antiviral activity against IAV [76]. The phosphorylation and mutation of Y20 lead to decreased IFITM3 ubiquitination [76]. IFITM3 can be monomethylated on the lysine 88 residue by lysine methyltransferase SET7, while the role of monomethylation in IFITM3’s antiviral activity remains unclear [86]. These multi-layered PTMs provide more means of regulating the activities of IFITMs under various physiological conditions.

## 4. The *ifitm* Genes in Different Species and Their Antiviral Ability

Although research on non-human IFITMs is limited, several recent studies reported the antiviral activity of IFITMs in bats and domestic animals [30,36,53,87,88,89]. In spite of duplication and polymorphisms in the *ifitm* genes, an alignment of 160 amino acid sequences of IFITM proteins in vertebrates demonstrates that the IFITM family members contain a conservative CD225 domain and two terminal hypervariable regions [5]. Several amino acid residues and motifs in the CD225 domain are central to the antiviral activity of IFITMs, such as the amphipathic helix [80,90,91], the cysteine residues for S-palmitoylation [77], and a GxxxG motif [75]. Both pig and microbat IFITM3 inhibit cell entry mediated by multiple IAV hemagglutinin and lyssavirus glycoproteins. Knockdown of IFITM3 in newborn pig trachea (NPTr) cells and primary microbat cells also enhance virus replication [88]. S-palmitoylation is critical for antiviral restriction by microbat IFITM3 and has a significant effect on its subcellular localization [87]. Functional studies have confirmed that codon 70, within the conserved CD225 domain of IFITMs in mammals, influences the antiviral activity and S-palmitoylation of microbat IFITM3 [87]. IFNs and/or viral infection induce the expression of canine IFITMs (caIFITM), which include caIFITM1, caIFITM2a, caIFITM2b, and caIFITM3. They have potent antiviral activity against the canine influenza virus [36]. Similarly, Cai et al. discovered that swine IFITM1a (swIFITM1a), -1b, -2, -3, or -5 can significantly inhibit the replication of African swine fever virus (ASFV), and knocking out these genes increased ASFV replication [53]. Additionally, chicken IFITMs (chIFITMs) serve various functions in different host cells. ChIFITM3 exhibits an antiviral role in chicken fibroblasts, and knocking down the constitutive expression of chIFITM3 in chicken fibroblasts enhances IAV infection [89]. Notably, chIFITM1 significantly increases IBV replication in the chicken hepatoma cell line LMH [30]. Since certain IFITM functions are conserved in different species, it is likely that IFITMs could serve as a barrier for virus cross-species spillover. For example, chimeric SIV/HIV-1 viruses (SHIVs), which have HIV-1 Env in the SIV genome, exhibit poor replication in macaque lymphocytes, and this restriction is partially attributed to the inhibition by macaque IFITM proteins [92]. Adaption of this SHIV to better replicate in macaque lymphocytes leads to changes in HIV-1 Env and resistance to macaque IFITMs, supporting the role of IFITMs in preventing early cross-species spillover events [92].

## 5. Mechanisms for IFITMs Inhibition of Membrane Fusion

**Figure 3:**
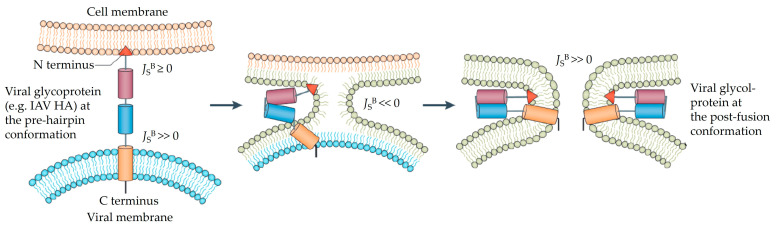
The process of viral and cell membrane fusion. The cell membrane (orange) and viral membrane (blue) are in close proximity, allowing the insertion of the fusion peptide at the N-terminus of the viral glycoprotein to the cell membrane. The energy generated from the viral glycoprotein refolding merges the viral and cell membrane. The outer leaflets mix and form hemifusion, followed by the inner leaflets merging and fusion pore formation. The bilayer spontaneous curvature values (J_s_^B^) of the cell and viral membranes are labeled. Both the viral and cell membranes are flat or positive curvatures (J_s_^B^ > 0 or J_s_^B^ >> 0). During the membrane fusion process, the curvature of both membranes transit from positive curvature to negative curvature at hemifusion (J_s_^B^ << 0) and recover to positive curvature after fusion pore formation (J_s_^B^ >> 0). The energy cost for this transition is determined by membrane rigidity and the radius of viral particle and endosome or plasma membrane where the membrane fusion occurs [93]. Figure adapted from Vigant et al. [93].

An important mechanism of IFITM restriction of virus infections is through modulating membrane properties. The fusion between cellular and viral membranes is essential for infection by enveloped viruses. Fusion of the viral and cellular membranes is a three-step process: (1) the membranes are brought to proximity where the repulsive electrostatic forces need to be overcome before lipids of the outer leaflets can interact; (2) the outer leaflets of the opposing membranes mix, resulting in hemifusion; (3) the inner leaflets of the lipid bilayers mix, opening a fusion pore (Figure 3) [93]. In some cases, the fusion pore opening is followed by an irreversible fusion pore expansion that allows the delivery of the viral contents into target cells [93]. The biophysical characteristics driving this process involve membrane fluidity facilitating lipid mixing alongside a notable shift from positive to negative membrane curvature [93]. These features depend on the lipid composition of both viral and cellular membranes. The energy required to overcome electrostatic repulsions between membranes and drive the transitions in membrane curvatures is supplied by conformational changes in viral glycoproteins upon binding to the host receptor or alterations in pH [93]. These essential features of the virus–cell fusion provide a framework for understanding the IFITM proteins’ role in restricting viral entry.

IFITMs can restrict membrane fusion by targeting hemifusion and fusion pore formation (Figure 3). For example, IFITMs block membrane hemifusion mediated by the Jaagsiekte sheep retrovirus envelope and IAV hemagglutinin, partly as this block cannot be rescued by chlorpromazine (CPZ), a chemical known to promote the transition from hemifusion to full fusion, but can be counteracted by oleic acid, which induces negative membrane curvature and promotes hemifusion [14] (Figure 3). More prominently, in situ cryo-electron tomography captures IFITM3-mediated arrest of IAV membrane fusion in endosomes, supporting that IFITM3 stabilizes hemifusion between the viral and endosomal membranes [16]. Further, single-virus imaging showed that IFITM3 overexpression abrogates the release of viral content into the cytoplasm by inhibiting pore formation, but does not inhibit lipid mixing, a hallmark of hemifusion formation [94], between the fluorescence-labeled IAV viral membrane and endosomal membrane [15]. Regardless, current evidence supports that IFITMs restrict viral–cellular membrane fusion by inhibiting the transitional steps at hemifusion and fusion pore formation.

**Figure 4:**
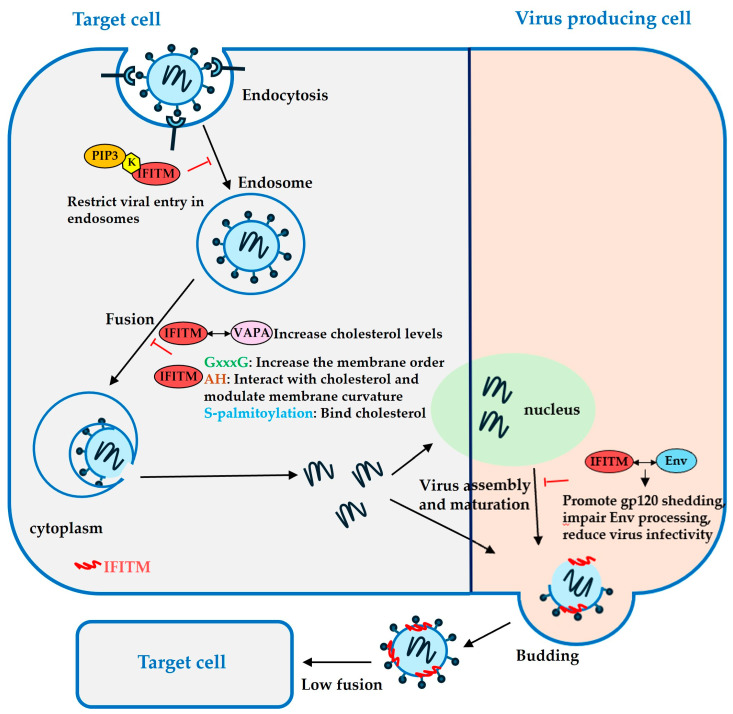
Mechanisms by which interferon-inducible transmembrane proteins (IFITMs) inhibit membrane fusion in target cells and the production of infectious viruses on viral-producing cells. IFITMs recruit 3,4,5-triphosphate (PIP3) through lysine residues in the CIL domain to restrict viral entry into endosomes. IFITMs modulate membrane fluidity and curvature by GxxxG motif, amphipathic domain, and S-palmitoylation. IFITMs incorporated into virions inhibit virus spread from virus-producing cells to new target cells.

IFITMs inhibit membrane fusion by modulating membrane fluidity and curvature, and this activity has been mapped to several motifs in IFITMs [75,80,91,95]. Amphotericin B (AmphoB) is an anti-fungal drug that binds to a fungal membrane constituent, ergosterol, leading to pore formation and ion egress [75,95]. AmphoB rescues IFITM2- and IFITM3- restrictions of IAV by counteracting IFITMs-mediated membrane order enhancement, as shown by fluorescence lifetime imaging [75]. Moreover, IFITM1 inhibits IAV HA-mediated cell–cell fusion by decreasing the membrane fluidity via fluorescence recovery after photobleaching assay (FRAP) [95]. The IFITM’s ability in membrane order and curvature modulation is conferred by an oligomerization motif GxxxG and an amphipathic helix (AH, Figure 2) domain, conserved among IFITM1, 2, and 3 (Figure 2). The GxxxG motif was found to be responsible for increasing the membrane order (Figure 4), thus rendering IFITM3 the ability to restrict IAV, as measured by Laurdan staining and FliptR [75]. The AH domain is known to generate membrane curvature via asymmetric leaflet expansion [96]. The IFITM3 AH partitions into lipid-disordered domains, induces negative membrane curvature in liposomes, and increases lipid order and membrane stiffness, as assessed by Laurdan staining [80]. The membrane modulation ability of the IFITM3 AH peptide correlates with the fusion inhibition activity, as targeting the ectopically expressed AH peptide to the cytoplasmic leaflet of the cell plasma membrane diminishes IAV-cell surface fusion induced by exposure to acidic pH [80,90].

The IFITM-mediated modulation of membrane fluidity and curvature may be attributed to its ability to undergo lipid sorting [16,91,96]. IFITM3 can interact with cholesterol directly via the AH domain, and disruption of the AH structure inhibits the cholesterol binding and membrane insertion of IFITM3 in cultured cells [91]. IFITMs have also been shown to bind cholesterol via S-palmitoylation, and the antiviral activity of IFITMs is correlated with the level of S-palmitoylation [79]. IFITM3 recruits phosphatidylinositol 3,4,5-triphosphate (PIP3) via the lysine residues in the CIL domain [97,98] (Figure 4). These lysine residues are required for IFITM3 restriction of viral entry in endosomes not at the plasma membrane [97]. Continuum modeling of the IAV-endosome fusion site based on the theory of lipid membrane elasticity predicts that the palmitoylated IFITM3 induces the local depletion of cholesterol and recruitment of negative curvature lipids, leading to a decrease in mechanical stress and increased fusion pore tension, which, in turn, increases the energy barrier for fusion pore expansion and inhibits membrane fusion [16]. IFITMs have been reported to disrupt intracellular cholesterol homeostasis by interacting with vesicle–membrane–protein-associated protein (VAPA) and antagonizing VAPA-oxysterol-binding protein (OSBP) function [99]. The IFITM3-VAPA interaction results in increased cholesterol levels in multivesicular bodies and late endosomes. This is believed to block the infection of VSV and IAV by inhibiting viral release from the endosomes [99,100]. However, conflicting data cast a shadow on the role of VAPA in IFITM-mediated viral restriction. A study shows that IFITM3 overexpression poorly correlates with the total cellular cholesterol, and the LDL-derived cholesterol transport inhibitor and knockdown of the cholesterol transporter NPC1 do not inhibit the membrane fusion of IAV and VSV [15]. Another study shows that overexpression of VAPA and disruption of the VAPA–IFITM3 interaction by mutations modestly alleviate IFITM3 restriction of IAV infection [95]. Overall, these studies suggest that IFITMs participate in lipid sorting and thus modulate membrane curvature and fluidity at the local virus entry sites.

## 6. IFITMs Inhibit the Production of Infectious Viruses in Infected Cells

IFITMs are expressed in both virus target cells and virus producer cells. While protecting virus target cells from being infected, IFITMs in virus producer cells also exert their antiviral activity by getting incorporated into viral progeny and negatively affecting viral infectivity and spread [101]. Studies reveal that IFITMs impair HIV-1 Env processing, especially IFITM2 and 3, by directly interacting with Env in virus-producing cells and promoting gp120 shedding [11]. Interestingly, all three IFITM proteins expressed in virus-producing cells are incorporated into HIV-1 virions, but the restriction activity does not correlate with their abundance in the virions, suggesting that the membrane fusion restriction by IFITMs on virions is independent of their incorporation levels [11]. A later study confirms that IFITM2 and IFITM3 in virus-producing cells reduce virus infectivity by causing defective processing of Env and degradation of Env precursor in endolysosomes, wherein decreased Env levels in IFITM2- and IFITM3-positive virus-producing cells are also observed [102] (Figure 4). This antiviral function of IFITM3 in virus producer cells can be overcome by the glycoGag protein, a murine leukemia virus (MLV) protein previously known to antagonize the antiviral activity of serine incorporator (SERINC) proteins, as well as by a high abundance of Env protein in virions [102]. However, HIV-1 Env defect is not evident in IFITM-positive virions that are produced by U87 cells expressing physiological levels of IFITMs, and that the IFITM restriction on HIV-1 infection is mainly conferred in target cells [103]. Further highlighting the importance of IFITMs in virus producer cells in restricting viral infection, it is reported that IFITM3 in uninfected target cells does not inhibit HIV-1 infection when infection is mediated by co-cultured HIV-infected lymphocytes, while overexpression of IFITM3 in virus-producing lymphocytes and 293T cells restrict viral spread [101]. Moreover, the expression of IFITM2 and IFITM3, but not IFITM1, in the virus-producing 293T cells significantly inhibited the spread of HIV-1 to Jurkat or HeLa cells [11]. This difference was mapped to the C-terminus of the IFITM proteins using a series of chimeric IFITM mutants [11]. Together, these data suggest that IFITMs are more effective in preventing cell-to-cell transmission of HIV-1 when present in virus producer cells than in virus target cells [11]. Nonetheless, IFITMs were found to become incorporated into a variety of different viruses and diminished viral infectivity, supporting the mechanism of IFITMs’ action against viral progeny [50].

## 7. Viral Glycoproteins Regulate the Viral Sensitivity to IFITMs

Not all viruses are inhibited by IFITMs. Early studies already reported that glycoproteins of Lassa virus (LASV) and MLV, when used to pseudotype lentivirus particles, confer resistance to IFITMs [3]. A recent study showed that LASV-pseudotyped virus particles, after being endocytosed, are able to avoid fusion with IFITM3-positive membrane compartments, whereas IFITM3-sensitive viruses, such as IAV and EBOV-pseudotyped viruses, are intercepted by these IFITM3-positive compartments [19], supporting the scenario that LASV glycoprotein evades IFITMs by directing and completing virus entry at an intracellular site devoid of IFITMs. Some viruses have even developed a dependence on IFITMs. For example, infection of common cold human coronavirus OC43 is promoted by IFITM2 and IFITM3 at the step of virus entry [26]. Knockdown of IFITM proteins severely impairs SARS-CoV-2 replication [17]. Mechanistic studies of this pro-viral effect are expected to shed more light on the complex functions of IFITMs in viral infections.

For viruses that are restricted by IFITMs, different viral strains can exhibit various sensitivities. One prominent example is HIV-1. Transmitted founder HIV-1, which initiates HIV-1 infection, was found to resist IFITM inhibition but become sensitive over time as mutations accumulate in viral Env glycoprotein in order to escape from neutralizing antibodies [103]. This observation was confirmed by subsequent studies reporting that HIV-1 Env clones from the transmitted founder HIV-1 strains are resistant to IFITM3, but the resistance is gradually lost as infection progresses into the acute and chronic stages [104] and that the increased IFITM sensitivity results from the acquisition of mutations in Env and Vpu, which mediate escape from neutralizing antibodies [105]. Interestingly, the IFITM3-sensitive HIV-1 is more inhibited by the neutralizing antibody PG16 than the IFITM3-resistant viruses, suggesting that the IFITM3 in virus producer cells may modulate the conformation and the degree of epitope exposure of viral Env [105]. Further studies mapped the IFITM3 resistance of an HIV-1 strain AD8-1 to the V3 loop on Env [106], which is corroborated by the results of a separate study showing that the IFITM3 resistance of HIV-1 clones is conferred by the V1, V2, and V3 loops in viral Env [105]. The IFITM3-resistant Env is believed to adopt a neutralizing antibody-resistant, low-energy, and “closed” conformation [106]. These studies suggest that HIV-1 Env glycoprotein is under the selection pressures of both IFITMs (innate immunity) and neutralizing antibodies (adaptive immunity); the necessity of evading one may increase sensitivity to the other.

Development of viral resistance to IFITMs has been recapitulated in long-term replication of HIV-1 in IFITM-expressing CD4+ T cells, and the resistant mutations were mapped to viral Env glycoprotein, further confirming the role of Env in assisting the escape from IFITM restriction. For example, an IFITM1-sensitive HIV-1 strain BH10 was able to turn resistance by mutating viral Env at G367E and inserting a stop codon at amino acid position 34 of Vpu [107]. Similarly, exchanging the Env sequences between the IFITM1-sensitive BH10 and IFITM1-resistant NL4-3 reversed the susceptibility of the parental strains to IFITM1 inhibition [108]. Mechanistic studies revealed that the mutated Env and the loss of Vpu increase cell-to-cell transmission of HIV-1, and this gain-of-function resists IFITM1 inhibition, thus warranting efficient spread of HIV-1 among cultured cells [11,107].

IFITM1, 2, and 3 assume different subcellular localization, with IFITM2 and 3 predominantly localized to endosomal and lysosomal compartments, whereas IFITM1 is more observed at the plasma membrane. The outcome is that all three IFITM proteins together guard virus entry sites both at the cell surface and the intracellular membrane compartments if all three proteins are equally well expressed in a given cell type. Because of the distinct subcellular localization of IFITMs, viruses, depending on their primary entry sites, exhibit different sensitivity to each of the three IFITM proteins. In a similar vein, a virus may avoid a potent inhibitory IFITM protein by altering its entry route. For example, HIV-1 glycoprotein, upon binding to receptor CD4, can use either CCR5 or CXCR4 as a co-receptor to complete entry, and co-receptor usage can dictate the site of viral entry. It was reported that the CXCR4 HIV-1 displays significantly greater sensitivity to IFITMs 2 and 3 than the CCR5 HIV-1, while most CCR5-using viruses were more sensitive to IFITM1 than CXCR4-using viruses [103]. The swap of the co-receptor usage determinants at the V3-loop of the Env protein results in exchange for the IFITMs restriction phenotypes of the viruses [103]. These findings suggest that the Env glycoprotein dependent-entry route of HIV-1 modulates viral sensitivity to IFITMs. This report is contested by the observations of a separate study showing that both CCR5 and CXCR4 HIV-1 strains are equally restricted by the three IFITM proteins in both lymphocytes and monocytes [109]. More studies are warranted to address this controversy.

Similarly, the sensitivity of SARS-CoV-2 to IFITM inhibition is modulated by the protease TMPRSS2 usage and virus entry route. For example, the cellular protease furin cleaves the polybasic cleavage site between the S1 and S2 subunits in the Spike (S) protein. This cleavage enables SARS-CoV-2 entry at the cell surface where the protease TMPRSS2 is expressed and cleaves the S protein at the S2’ site, leading to the exposure of fusion peptide and initiating membrane fusion [20]. Mutating this polybasic site in the S1/S2 boundary renders virus entry to endolysomes, and the mutated virus is much more inhibited by endosomal IFITM2 protein [80,90]. Along with SARS-CoV-2 evolution, more mutations are accumulated in the S protein to escape from neutralizing antibodies that are produced either in response to natural SARS-CoV-2 infection of COVID-19 vaccines. The omicron variant accumulates so many mutations in the S protein that its entry becomes TMPRSS2-independent and follows the endocytic pathway [110]. As a result, omicron SARS-CoV-2 is more inhibited by IFITM2 and IFIM3 than the earlier variants, including Beta and Delta [25]. This presents an example further illuminating the concept that viral escape from the adaptive immunity pressure only sensitizes the virus to innate immunity, which is expected to contribute to viral attenuation.

## 8. Immune Modulation by IFITMs

IFITMs play important roles in the regulation of adaptive and innate immune responses. Stacey et al. used murine cytomegalovirus (CMV) as a model to show that IFITM3 limits herpesvirus-associated pathogenesis without directly preventing virus replication but by regulating antiviral cellular immunity that controls cytokine-driven viral pathogenesis [60]. IFITMs also regulate murine CD4+ Th cell differentiation, thus influencing Th1/Th2 polarization in allergic airway disease models [111]. IFITMs regulate T and B cell differentiation and Th2 inflammatory activity as transcriptional targets of Bcl6 and hedgehog/Wnt signaling pathway [112,113,114,115]. On a cellular level, IFITM3 fine-tunes the response of myeloid cells to CMV infection by promoting the proteasomal degradation of the reticulon 4 isoform, Nogo-B. Nogo-B mediates TLR-dependent pro-inflammatory cytokine production, elevates viral pathogenesis in vivo, and alters the cellular localization of TLR2 and TLR2 responses [116]. An in-depth mechanistic study showed that IFITM3 was responsible for the malignant transformation of B cells by forming a PIP3 scaffold to promote PI3K signaling amplification downstream of B cell receptors and adhesion receptors. IFITM3-mediated assembly of the signaling complex leads to the recruitment of B cell receptor, integrin receptors, and cholesterol and the amplification of PI3K signaling downstream of the BCR and adhesion receptor [98]. IFITM3 has also been shown to associate with IRF3, a key transcription factor regulating the expression of IFNs, and regulate the homeostasis of IRF3 by mediating autophagic degradation of IRF3 [117]. Similarly, IFITM3 may regulate the cGAS-STING-IRF3 signaling pathway by directly binding to STING and p62/SQSTM1, which participate in a negative feedback loop on the cytosolic DNA sensing pathway by facilitating autophagic degradation of STING [118,119,120]. Another study showed that IFITM1/3 promotes the IFNγ-stimulated synthesis of HLA-B and ISG15 in cervical cancer cells, thus leading to enhanced expression of MHC Class I molecules and antitumor innate immune response [121]. These studies highlight the pivotal role of IFITMs in immune regulation, orchestrating a broad spectrum of antiviral responses by modulating both innate and adaptive immunity, including the differentiation of T and B cells, and influencing cytokine-driven pathways and cellular signaling mechanisms. This multifunctional involvement underscores their critical function in fine-tuning the immune system’s balance between antiviral defense and inflammatory pathogenesis.

## 9. Conclusions

As a key player in innate immunity, IFITMs act as the first line of defense against viral infection. Studies in the past two decades have established that IFITMs restrict virus replication of enveloped viruses by affecting membrane fluidity and curvature and, as a consequence, membrane fusion. IFITMs, especially IFITM3, have been shown to play multifaceted roles in regulating the various signaling pathways involved in both adaptive immunity and cancer development [122,123]. Despite the conserved ability in membrane property modulation, the antiviral activity of IFITMs depends on the post-translational modification [6], the virus entry routes [25], the virus strains, and viral glycoproteins [11,103,106], the host factors [27], and the cell types [17]. Given the context-dependent nature of IFITMs’ antiviral activity, it is critical to broaden the investigation and interpretation of their mechanisms beyond the localized virus attachment site. Instead, it is essential to consider how the interplay between host factors and IFITMs, triggered by virus infection, leads to broader changes in antiviral response in infected cells, tissue, and/or individuals. Moreover, studies on HIV-1 and SARS-CoV-2 have revealed that the IFITM sensitivity conferred by the viral glycoprotein, links to the selection pressures imposed by adaptive immunity. The delicate equilibrium between viral evasion of host immunity and viral transmission is expected to shape the evolution of existing viruses and the emergence of new ones. Understanding the precise mechanisms governing this balance is crucial for elucidating and predicting virus pathogenesis and prevalence.

## Figures and Tables

**Table 1:** Viruses targeted by IFITMs and potential mechanisms of action.

Viruses Targeted	Mechanisms of Action	Subcellular Localization	References
***RNA viruses***			
*Coronaviridae*			
Severe acute respiratory syndrome coronavirus 2 (SARS-CoV-2)	SARS-CoV-2 spike (S) hijacks IFITM2 for efficient infection by interacting with ACE2.	Plasma membrane, endosomes	[17,21,22]
	IFITMs Inhibits SARS-CoV-2 S-mediated membrane fusion and impairs cell surface expression of ACE2.	Endosomes, plasma membrane	[18,23,24,25]
Severe acute respiratory syndrome coronavirus (SARS-CoV)	IFITMs inhibit SARS-CoV S-mediated entry, and the inhibition can be counteracted by trypsin treatment, which bypasses the SARS-CoV’s dependence on lysosomal cathepsin L.	Lysosomes	[9]
Human coronavirus OC43 (hCoV-OC43)	HCoV-OC43 uses IFN-induced IFITM2 or IFITM3 as an entry factor to facilitate its infection in host cells.	Endosomes	[26]
Human coronavirus 229E (hCoV-229E)	IFITMs inhibit hCoV-229E S-dependent entry. The inhibition can be rescued by TMPRSS2 treatment.	Endosomes	[27]
Middle East respiratory syndrome coronavirus (MERS-CoV)	IFITMs inhibits MERS-CoV entry by a cholesterol-independent mechanism.	Endosomes	[28]
Transmissible gastroenteritis virus (TGEV)	IFITM3 inhibits TGEV replication and interferes with its binding to PK15 cells.	Endosomes	[29]
Coronavirus infectious bronchitis virus (IBV)	The antiviral effects of chIFITMs on IBV depend on virus and cell types.	Cell membrane	[30]
*Retroviridae*			
Human immunodeficiency virus type 1 (HIV-1)	IFITMs restrict HIV-1 infection by antagonizing HIV-1 envelope glycoprotein (Env), modulating membrane property, reducing viral particle infectivity via incorporation into virus particles, and inhibiting viral protein synthesis by excluding viral mRNA from polysomes.	Cell surface, endosomes	[11,12,31,32]
Simian immunodeficiency viruses (SIV)	IFITMs inhibit the cell entry of SIV, and the inhibition depends on the viral vectors and incorporation of SIV-Env.	Cell surface, endosomes	[33]
Foamy virus (FVs)	IFITM1-3 overexpression inhibits prototype FV entry into target cells and reduces the number of prototype FV envelope proteins.	Plasma membrane, intracellular compartments	[34]
Feline foamy virus (FFV)	IFITMs inhibit FFV at late steps of viral replication.	Intracellular membranes, plasma membrane	[35]
*Orthomyxoviridae*			
Canine influenza virus (CIV)	caIFITM1, caIFITM2a, caIFITM2b, and caIFITM3 inhibit the fusion of viral and cellular membranes.	Cell surface and cytoplasm	[36]
Influenza A viruses (IAV)	IFITM1, 2, and 3 restrict an early step in IAV replication by blocking the fusion pore enlargement and modulating membrane properties.	Late endosomes	[3,15,16]
*Paramyxoviridae*			
Parainfluenza virus-3 (PIV-3) Metapneumovirus (MPV) Respiratory syncytial virus (RSV)	IFITM1 blocks the fusion between viral and cellular membranes.	Plasma membrane	[37]
Nipah virus (NiV)	IFITM3 promotes NiV glycoproteins-mediated virus entry by interacting with the fusion protein.	Plasma membrane and endosome	[38]
*Flaviviridae*			
Tick-borne encephalitis virus (TBEV)	IFITM1, IFITM2, and IFITM3 inhibit TBEV infection and prevent virus-induced cell death.	Plasma membrane, endosomal membrane, and lysosomal membranes	[39]
Dengue virus (DENV) West Nile virus (WNV)	IFITM2 and IFITM3 disrupt early steps of the viral infection.	Endosomes	[40]
Hepatitis C virus (HCV)	IFITM1 disrupts HCV viral entry by interruption of viral co-receptor functions at the tight junctions of HCV-infected liver cells.	Tight junction, cell surface	[41]
Zika virus (ZIKV)	IFITM1 and IFITM3 inhibit ZIKV infection early in the viral life cycle. IFITM3 can prevent Zika virus-induced cell death.	late endosomes and lysosomes	[13]
Yellow fever virus (YFV)	IFITMs, especially IFITM3, block membrane fusion by toughening the host membrane.	Late endosomes	[3]
*Rhabdoviridae*			
Vesicular stomatitis virus (VSV)	IFITMs inhibit virus infection by blocking virus fusion with cell membranes.	Plasma membrane, endosomes	[2,42]
*Filoviridae*			
Ebolavirus (EBOV) Marburg virus (MARV)	IFITMs inhibit the replication of infectious EBOV and MARV. EBOV and MARV are more susceptible to IFITM1 than IFITM3.	Lysosomes	[9]
*Reoviridae*			
Rotavirus (RV)	IFITM3 limits RV infection by affecting the function of the late endosomal compartment.	Late endosome	[8]
*Arteriviridae*			
Porcine reproductive and respiratory syndrome virus (PRRSV)	IFITM3 inhibits PRRSV by inducing cholesterol accumulation and impairing viral-cell membrane fusion.	Early endosomes, late endosomes, and lysosomes	[43]
*Togaviridae*			
Chikungunya virus (CHIKV)Mayaro virus (MAYV)	IFITMs restrict CHIKV and MAYV infection at glycoprotein-mediated entry, both in the context of direct infection and cell–cell transmission.	Cell surface and endosomes	[44]
Sindbis virus (SINV) Semliki Forest virus (SFV)	IFITMs, especially IFITM3, restricts SINV and SFV by inhibiting virus–cell membrane fusion.	Early endosomes	[45]
*Bunyaviri* *ales*			
Rift Valley fever virus (RVFV) La Crosse virus (LACV) Hantaan virus (HTNV) Andes virus (ANDV)	IFITM2 and 3 prevent virus membrane fusion in the endosomes.	Endosomes	[46]
***DNA viruses***			
*Herpesviridae*			
Kaposi’s Sarcoma-associated herpesvirus (KSHV) and Rhesus Monkey Rhadinovirus (RRV)	IFITM1 restricts KSHV and RRV by acting at the level of membrane fusion.	Cell surface	[47]
Pseudorabies virus (PRV)	IFITMs restrict PRV infection by interfering with PRV cell binding and entry. IFITM2 inhibits PRV by regulating cholesterol levels in endosomes.	Endosomes	[48]
Herpes simplex virus-1 (HSV-1)	IFITM1 restricts infection of HSV-1 that enters at the plasma membrane.	Plasma membrane	[37]
Human Cytomegalovirus (HCMV)	HCMV exploits IFITMs to facilitate the formation of the virion assembly compartment in human fibroblasts.	Cytoplasm	[49]
Epstein–Barr virus (EBV)	IFITM1 enhances the initial entry of EBV, but the incorporation of IFITM2/3 into viral particles reduces the infectivity of progeny viruses.	Cytoplasm	[50,51]
*Poxviridae*			
Vaccinia virus (VACV)	IFITM3 restricts VACV infection, replication, and proliferation by interfering with virus entry processes prior to virus nucleocapsid entry into the cytoplasm. VACV counteracts IFITM3 by inhibiting its translation.	Endosomes	[52]
*Asfarviridae*			
African swine fever virus (ASFV)	SwIFITM1a, -1b, -2, -3, or -5 restrict the fusion of virus membrane and plasma membrane.	Plasma membrane, cytoplasm, and the perinuclear region.	[53]

## Data Availability

Not applicable.
